# Osteochondroma of the Mandible: A Rare Case Report

**DOI:** 10.1155/2013/167862

**Published:** 2013-09-26

**Authors:** Donepudi Nanda Kishore, H. R. Shiva Kumar, K. V. Umashankara, Kirthi Kumar Rai

**Affiliations:** Department of Oral, Maxillofacial and Reconstructive Surgery, Bapuji Dental College and Hospital, Rajiv Gandhi University of Health Sciences, Davanagere, Karnataka 577004, India

## Abstract

Osteochondroma, also known as osteocartilaginous exostosis is a benign cartilage forming tumor that usually develops in long bones and relatively uncommon in the craniofacial region. Both the condyle and coronoid tip being the most common sites of occurrence in the mandible, it rarely appears at the symphysis region. Here, we describe a case of osteochondroma arising from the left parasymphysis of mandible.

## 1. Introduction

Osteochondroma is an osseous protuberance with cartilaginous growth potential that usually appears near the growth plate at the ends of long bones such as knee, hip, shoulder, and joints. Only about 1% of these occur within the head and neck region [1]. The most commonly occurring sites in the craniofacial region are the condyle and the coronoid process. These osteochondromas occurring in the mandibular symphysis region is extremely rare, and to our knowledge only two cases have been reported previously [2, 3]. Here, we describe a case of osteochondroma arising from the left parasymphysis of mandible.

## 2. A Case Report

**Figure 1:**
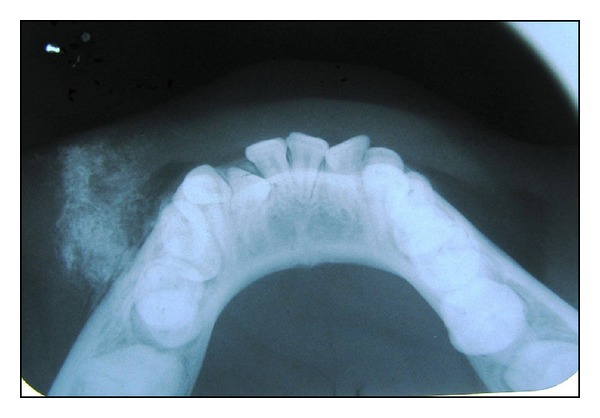
Preoperative occlusal radiograph revealed irregular radiopacities extending into soft tissue shadow of buccal mucosa and lower lip from the buccal cortex.

A 9-year-old female child was referred to our maxillofacial unit because of a painless bony hard swelling in the left parasymphysis of mandible. The child's mother had noticed this swelling several years ago but did not seek any medical attention. Patient had no history of trauma. Bilateral submandibular lymph nodes were palpable. An extraoral swelling of 2 × 2 cm size was evident at the corner of the mouth. On clinical palpation, well-defined, irregular bony hard, nontender mass was felt which is fixed to the underlying buccal cortical plate. The overlying skin appears slightly fixed. Occlusal radiographic view revealed an irregular multiple foci of radiopacity merging with buccal cortex and extending into soft tissue shadow of buccal mucosa and lower lip (Figure 1). Internal structure reveals crops of radiopacity and radiolucency. The USG report shows diffuse hyperechoic areas with irregular margins extending from mental region to 2 cm towards lateral side. Provisional diagnosis of osteoma was established. The routine blood investigation was shown to be normal, except increase in the serum alkaline phosphatase level of 216%.

**Figure 2:**
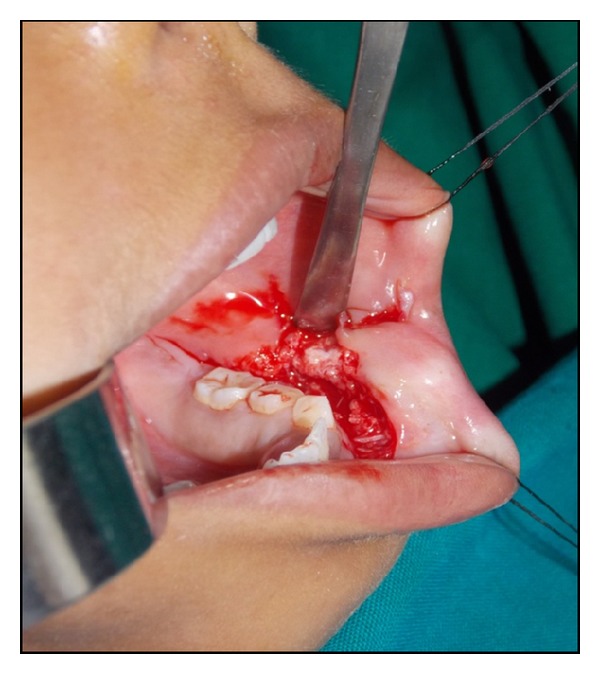
Intraoperative picture showing exposure of bony mass.

**Figure 3:**
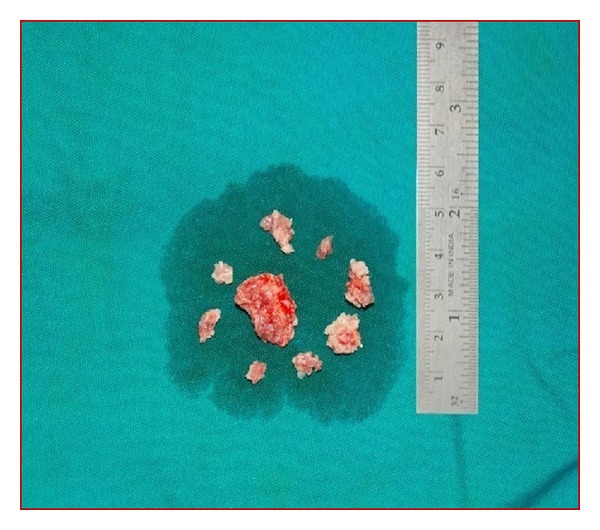
Bits of specimen for histopathological examination.

The clinical diagnosis that lurked in our minds was osteoma cutis, and the tumor excision was done under general anaesthesia. The mass was approached through labiobuccal vestibular incision. Intraoperatively, the bony mass was exposed and the dissection was limited to the base of the lesion. The irregular surface of the lesion was found to be grown into the buccal mucosa (Figure 2), which was cut into pieces and removed from the soft tissue. The mental nerve was exposed and preserved carefully. The basal attachment of this bony mass to the buccal cortical plate at the region of mental foramen was also sheared off as one large bit using surgical bur. The resected surface of the buccal cortical bone was rasped smooth, and the bits of specimen (Figure 3) were sent for histopathological examination.

**Figure 4:**
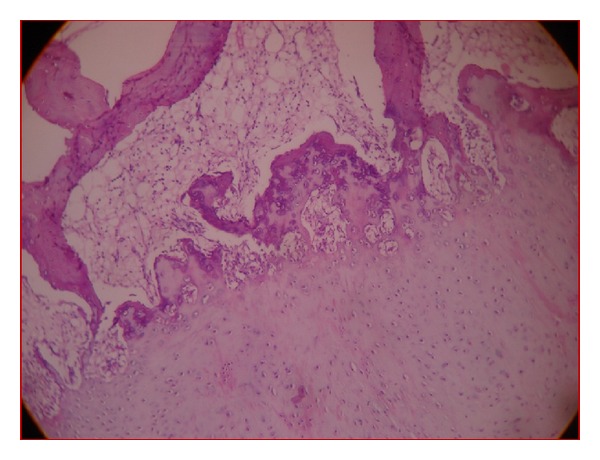
Microscopic view showing trabecular bone covered with a cartilaginous cap and columns of chondrocytes. Fatty connective tissue marrow is also seen (hematoxylin and eosin stain 10x).

Histopathological examination (Figure 4) revealed an osteochondroma. Cartilaginous cap with columns of chondrocytes and islands of cartilage growing into the trabecular bone with osteoblastic rimming and fat marrow is seen histopathologically.

## 3. Discussion

Osteochondroma forms as an exophytic growth from the surface of the affected bone [4]. It commonly arises in bones formed by endochondral ossification and rarely occurs in craniofacial bones, as these are formed by intramembranous ossification. The exact cause of this osteocartilaginous growth is still unknown. Historically, there has been debate whether these represent a developmental aberration or a benign tumor. The mandible has cartilage precursors in the regions of condyle and coronoid and on either side of symphysis [5]. In the mental region, one or two cartilages appear and ossify in the 7th month after-conception and form mental ossicles, which become incorporated into the intramembranous bone [5]. Residues of these cartilages appear to give rise to osteochondromas of this region [5]. Osteochondromas of jaws demonstrate a slight female predominance and affect older individuals, than do lesions of the long bones which are mainly seen in younger individuals [4]. The lesion in our case is more sessile type with flat and broad base than long, pedunculated. The bony mass was found attached to buccal cortical bone at mental foramen region and had grown towards inferior border of mandible rising into buccal mucosa like a cauliflower shape (Figure 2) indicating a soft tissue calcification underneath skin, giving rise to an impression of osteoma cutis. It is because of this type of growth, slight vestibular depth was still evident clinically. Intraoperatively, the lesion was found to be nonencapsulated, and the bony mass was piecemealed and excised. It was due to this removal of bits of mass from the soft tissue, postoperative radiograph revealed minute radiopacities. The mesenchymal membranous calcification and the absence of cartilaginous precursor in osteoma cutis differentiate with that of an osteochondroma. Osteochondromas are usually asymptomatic and symptoms may develop as consequences of size, impingement on adjacent structures such as nerves, or fracture through the stalk causing pain [6]. Local anatomic constraints must be considered carefully so that the approach and resection do not damage nearby structures. Intraoperatively, there is an impingement of mental nerve with that of the bony mass, and the underlying buccal cortex is continuous with the cortex of the bony growth. Histologically, these are characterized by the presence of a cartilaginous cap covering the tumor. With time, this cartilage is gradually replaced by bone. Here in our case, histologically, the bony trabeculae was covered with a cartilaginous cap, and bone marrow showed signs of fatty degeneration. Another differential diagnosis which can be thought of is osteocartilaginous choristoma, which commonly occurs on lateral border of tongue and rarely reaches a size of more than 1.5 cm [1].

Radical excision including surrounding periosteum is strongly recommended and recurrence is rare [7]. The resection is curative in almost all cases, provided that the entire lesion with its periosteal membrane is removed. These show low risk of recurrence or sarcomatous changes. The incidence of malignant change in multiple osteochondromatosis is higher than that in the solitary form (with 2–25% in multiple osteochondromatosis and 1–4% in solitary form) [4, 8]. Individuals with multiple osteochondromas show bone length discrepancy in long bones. Recurrences of osteochondromas affecting both long bones and jaws are only rarely reported [4].

## 4. Conclusion

Though osteochondromas are rare to occur in craniofacial bones, these bony lesions should sometimes lurk in the surgeons mind. Clinically, the presence of stalk like cortex continuous with the cortical bone suggests a true osteochondroma. These have tendency to grow parallel to the growth of the underlying parent bone as the patient's age advances. These may be asymptomatic initially, but mechanical irritation and cosmetic deformity by an underlying exostosis frequently lead to surgical resection. Care is required to ensure that the resection neither violates normal host cortex by staying too deep nor leaves residual lesion by staying too shallow. The overlying bursa should be left intact, and the loose adhesive tissue should be dissected away, so that the bursa and the lesion are removed en bloc. As long as the entire cartilaginous cap or perichondrium is removed there should be no recurrence. Although the outcomes of treatment and prognosis are good, it is advisable to follow up these cases, as recurred cases have also been reported. 

## 5. Future and Controversies

 Recent genetic karyotyping studies suggest that they may represent a true neoplastic process and not a reactive one. Research is in early stage and further investigation is needed. As the bone length discrepancies are seen in some cases of long bone multiple osteochondromas, it is quite fair enough to keep a periodic record of length of the affected mandible mainly when it is seen in younger individuals.
